# Exploring the Effects of Human Bone Marrow-Derived Mononuclear Cells on Angiogenesis In Vitro

**DOI:** 10.3390/ijms241813822

**Published:** 2023-09-07

**Authors:** Judith A. H. M. Peeters, Hendrika A. B. Peters, Anique J. Videler, Jaap F. Hamming, Abbey Schepers, Paul H. A. Quax

**Affiliations:** 1 Department of Surgery, Leiden University Medical Center, 2300 RC Leiden, The Netherlands; a.h.m.peeters@lumc.nl (J.A.H.M.P.); h.a.b.peters@lumc.nl (H.A.B.P.); anique.videler@hotmail.com (A.J.V.); j.f.hamming@lumc.nl (J.F.H.); a.schepers@lumc.nl (A.S.); 2 Einthoven Laboratory for Experimental Vascular Medicine, Leiden University Medical Center, 2300 RC Leiden, The Netherlands

**Keywords:** peripheral artery disease, chronic limb-threatening ischemia, angiogenesis, bone marrow-derived mononuclear cells, arteriogenesis, cell therapy

## Abstract

Cell therapies involving the administration of bone marrow-derived mononuclear cells (BM-MNCs) for patients with chronic limb-threatening ischemia (CLTI) have shown promise; however, their overall effectiveness lacks evidence, and the exact mechanism of action remains unclear. In this study, we examined the angiogenic effects of well-controlled human bone marrow cell isolates on endothelial cells. The responses of endothelial cell proliferation, migration, tube formation, and aortic ring sprouting were analyzed in vitro, considering both the direct and paracrine effects of BM cell isolates. Furthermore, we conducted these investigations under both normoxic and hypoxic conditions to simulate the ischemic environment. Interestingly, no significant effect on the angiogenic response of human umbilical vein endothelial cells (HUVECs) following treatment with BM-MNCs was observed. This study fails to provide significant evidence for angiogenic effects from human bone marrow cell isolates on human endothelial cells. These in vitro experiments suggest that the potential benefits of BM-MNC therapy for CLTI patients may not involve endothelial cell angiogenesis.

## 1. Introduction

Peripheral arterial disease (PAD) is a chronic condition where peripheral blood flow is restricted due to stenosis or blockage of the arteries. In an advanced state, PAD can lead to chronic limb-threatening ischemia (CLTI), resulting in patients suffering from rest pain and/or ischemic ulcers or gangrene. The current treatment for CLTI is directed at restoring the blood flow to the limb with endovascular or surgical interventions, in addition to standard drug therapy and cardiovascular risk management. Unfortunately, the success and patency rates of these interventions are around 60%. Due to the severity of the disease and shortcomings of current therapies, there is a need for new effective therapies.

In the last decades, the interest in the field of cell therapy, including stem cells, is rising, since this could be a promising alternative to conventional therapy. Cell therapy came to light early this century in 2002, when the first clinical study reported that bone marrow-derived mononuclear cells (BM-MNCs) could be safe and effectively used to treat CLTI [1]. Due to their potential ability to promote angiogenesis, BM-MNCs have been used in various clinical trials, showing beneficial effects for ulcer healing and limb salvage [2,3,4,5,6]. However, detailed analyses of various randomized controlled trials have failed to show clinically relevant beneficial effects [7]. Mononuclear cells are a mixture of different types of hematological cells, including lymphocytes, monocytes, and hematopoietic stem cells. They have been shown to have regenerative properties and the ability to promote angiogenesis [8,9]. However, the composition of cell therapies is largely variable, with various preparation methods and different routes of administration.

One of the promising new approaches is based on the use of REX-001, a highly standardized autologous bone marrow-derived mononuclear cell product that has shown significant blood flow recovery by increasing vascular density and functional neovascularization, which correlated with clinical benefits [10]. Due to these promising results, currently a phase III clinical trial is being conducted (ClinicalTrials.gov Identifier: NCT03174522). However, the exact mechanism of action of REX-001 is still unknown.

The neovascularization processes that lead to restoring the blood flow comprise both arteriogenesis and angiogenesis. Arteriogenesis is the recruitment of collaterals from pre-existing arterioles, and is mainly inflammatory driven. Angiogenesis is the formation of new capillary blood vessels, and plays a crucial role in various physiological and pathological processes involving the sprouting and remodeling of blood vessels from the pre-existing vasculature. The cell types that contribute to neovascularization are endothelial cells, circulating monocytes, smooth muscle cells, and pericytes [11,12]. Endothelial cells, which are important elements of blood vessels, play a pivotal role in angiogenesis. The proliferation and migration of endothelial cells are crucial events contributing to the formation of new vessels and the formation of a functional vascular network, and are driven by multiple growth factors and cytokines including vascular endothelial growth factor (VEGF), platelet-derived growth factor, insulin-like growth factor 1, interleukin 1, interleukin 6 (IL-6), and interleukin 8 (IL-8) [13,14]. In addition to proliferation, endothelial cell migration allows endothelial cells to navigate through the extracellular matrix to form new blood vessels. Endothelial cell migration is regulated by various signaling molecules, including VEGF and angiopoietins. Activated endothelial cells can release chemoattractants such as monocyte chemoattractant protein-1 (MCP-1), initiating the recruitment of monocytes to the angiogenic site [15,16,17]. 

Bone marrow-derived mononuclear cells (BM-MNCs) consist of a variety of cell types including lymphocytes, granulocytes, monocytes, and progenitor cells. It is hypothesized that BM-MNCs induce neovascularization, i.e., arteriogenesis and angiogenesis. In the current study the effect of BM-MNCs on angiogenesis was explored by studying endothelial cell proliferation, cell migration, angiogenic tube formation, and sprouting in different set-ups.

## 2. Results

### 2.1. Isolation and Quality Control of Bone Marrow-Derived Cells

**Figure 1:**
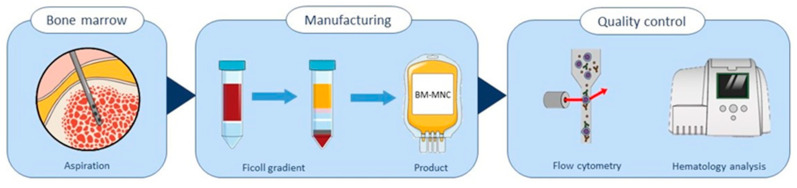
Illustration of the manufacturing process of BM-MNC isolates, starting with bone marrow aspiration, followed by manufacturing the product via Ficoll gradient cell separation, and finally a quality assessment was performed using flow cytometry and hematology analysis.

The bone marrow mononuclear cell isolates used in this study were obtained from healthy volunteers (Hemacare, Charles River, Wilmington, MA, USA) and isolated according to a strict protocol that met strict specifications, as defined by Rojas-Torres et al. [18]. The first step was to isolate the BM-MNC cells according this protocol. As shown schematically in Figure 1, the BM-MNCs were isolated from heparinized bone marrow via Ficoll gradient separation. The characteristics of the product are defined in Table 1.

The quality of the isolated BM-MNCs was analyzed using both hematology analysis and flow cytometry to demonstrate that the manufactured cell isolate met the quality acceptance criteria of the bone marrow cells isolates, as in the REX-001 clinical trial. The cell isolates in this study were produced according to the REX-001 manufacturing protocol.

Not all of the BM-MNC samples met the criteria of >15% leukocyte recovery; one sample only had 13.05% leukocyte recovery, and was not used in experiments (Table 1). All of the samples had >96% erythrocyte depletion and >60% thrombocyte depletion, meeting the quality criteria. Furthermore, all of the BM-MNC isolates met the following criteria: viability above 80%, containing >30% granulocytes, and the presence of CD34+/CD45+ cells (>0.1%).

In addition to the required quality assessment, a more extensive flow cytometry panel was used to characterize the cell composition of the BM-MNC isolates in more detail. The CD45+ cell fraction was analyzed further to determine the percentages of B lymphocytes and T lymphocytes. Subsequently, the T lymphocytes were further characterized to CD4+ and CD8+ T cells. In addition, the percentages of monocytes in the BM-MNC isolates were determined. The flow cytometry gating strategy is shown in Supplementary Figure S1.

### 2.2. BM-MNCs Have No Effect on Endothelial Cell Proliferation

To determine whether BM-MNCs have an effect on endothelial cell proliferation, directly or indirectly, human umbilical vein endothelial cells (HUVECs) were incubated either with increasing numbers of freshly isolated BM-MNCs or increasing concentrations of BM-MNC-conditioned medium, and the proliferation was analyzed with MTT assays. Based on previous experiments, the endpoints of both assays were set at 24 h after treatment with BM-MNCs.

**Figure 2:**
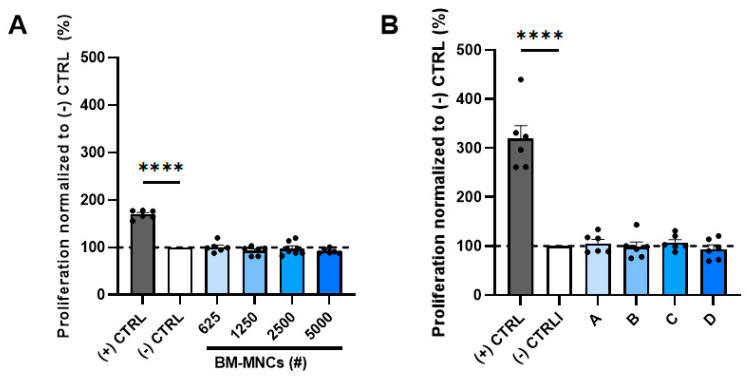
Quantification of HUVEC proliferation after treatment with either (**A**) BM-MNCs (625, 1250, 2500, or 5000 cells added indicated as BM-MNCs (#)) or with (**B**) BM-MNC-conditioned media A, B, C, and D, respectively, representative for 2500, 5000, 10,000, or 20,000 BM-MNCs. Graph 2A is representative for 6 experiments performed with BM-MNCs manufactured from 6 different bone marrow samples. Graph 2B is representative for 2 experiments performed with BM-MNC-conditioned media from 2 BM-MNC products. The positive control is EBM2 medium containing 2% serum, and the negative control is EBM2 medium containing 0.2% serum. Data are presented as mean ± SEM with datapoints (indicated as (●)) in sextuplicate. **** *p* ≤ 0.0001 via one-way ANOVA.

To explore a direct effect of BM-MNCs on endothelial cell proliferation, BM-MNCs were added directly to the HUVEC cultures. None of the doses of BM-MNCs tested (625, 1250, 2500, and 5000 cells) resulted in a change in HUVEC proliferation in the MTT assay as compared to the negative control, i.e., EBM2 medium with 0.2% serum. The proliferation was significantly lower than in the positive control group that was exposed to the EMB2 medium supplemented with growth factors. If any effect could be observed, this would be that with the higher BM-MNC dose, slightly less endothelial cell proliferation occurred (Figure 2A and Supplementary Figure S2A). The data shown in Figure 2A are from one representative experiment. All of the experiments with BM isolates for different donors showed a similar pattern, with no effects on HUVEC proliferation (Figure S2).

In addition to the direct effects on HUVEC proliferation by BM-MNC isolates, we studied whether proliferation could be induced by paracrine factors present in the isolate. For this investigation, we incubated HUVECs with increasing concentrations of BM-MNC-conditioned media. The concentration is defined as the equivalent of BM-MNC cells secreting their paracrine factors into the conditioned medium, representative for 2500, 5000, 10,000, or 20,000 BM-MNCs (Figure 2B).

To evaluate if the BM-MNCs would have an indirect effect on HUVEC proliferation in other conditions, conditioned medium was also prepared in media with less or more serum added. To evoke a potential effect, the assays were also executed in hypoxic conditions, since hypoxia induces vascular endothelial growth factor (VEGF), which is an angiogenic factor (Figure S3). Nevertheless, adding BM-MNCs in hypoxic conditions did not increase HUVEC proliferation. To evaluate whether the kind of culture medium led to different BM-MNC-conditioned medium with different effects on HUVEC proliferation, these experiments were also performed using immune cell-suitable culture media to optimize the culturing conditions for the BM-MNCs, OptiMEM, and AIMV, in order to prepare BM-MNC-conditioned medium (Figure S4). HUVEC proliferation after adding BM-MNC-conditioned medium in OptiMEM did not show any differences, whereas HUVEC proliferation after adding BM-MNC-conditioned medium in AIMV showed a decrease in HUVEC proliferation under hypoxic circumstances. Since in none of these conditions was any difference in HUVEC proliferation observed compared to the negative control, this suggests that BM-MNCs exert no paracrine effects on endothelial cell proliferation. 

### 2.3. BM-MNCs Do Not Affect Endothelial Cell Migration

**Figure 3:**
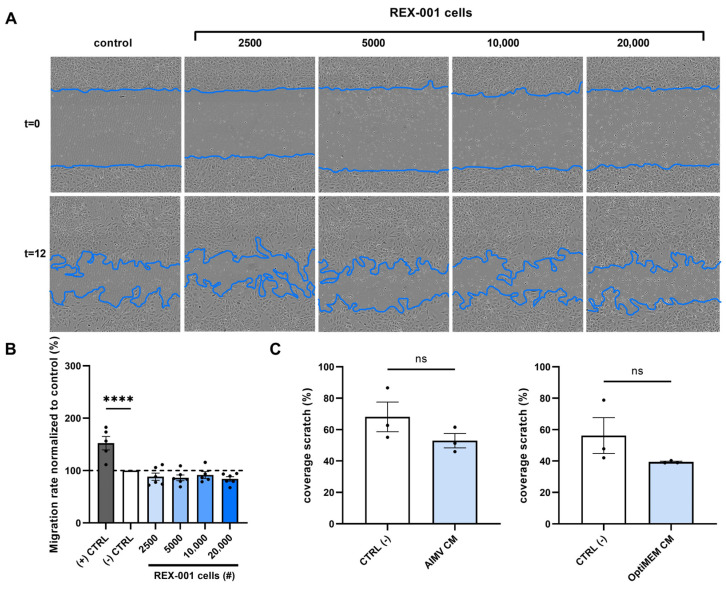
Representative pictures (**A**) and quantification of HUVEC scratch wound healing after (**B**) treatment with BM-MNCs (2500, 5000, 10,000, or 20,000 cells added) or (**C**) after treatment with BM-MNC-conditioned medium (AIMV or OptiMEM incubated with 1.33 × 10^6^ BM-MNCs/mL). Graphs (**B**) and (**C**) are representative for 6 and 2 experiments performed, respectively, with BM-MNC isolates manufactured from different bone marrow samples. Data points represent technical replicates, six and two, respectively, and are presented as mean ± SEM. ns = non-significant, **** *p* ≤ 0.0001 via one-way ANOVA.

Next to endothelial cell proliferation, endothelial cell migration is a key process involved in the formation of new vessels that might be stimulated by bone marrow cell isolates. To evaluate the effect of BM-MNCs on HUVEC migration, wound healing assays were performed. After culturing HUVEC for 24 h, a scratch wound was introduced into monolayers of HUVECs using the Incucyte Woundmaker Tool. Subsequently, these wounded cultures were treated with different doses of BM-MNCs. The plates were incubated in the IncuCyte S3, and pictures were taken after 12 h. The percentage of scratch-wound closure after 12 h was calculated. Figure 3A clearly shows the increasing concentration of BM-MNCs that was added at t = 0, visualized as cells over the wounded area.

Quantification of the scratch wound closure rate of HUVECs treated with BM-MNCs was performed after 12 h. The experiments were performed in six-fold with different BM-MNC isolates, and the results did not show an increase in migration rate (Figure 3B and Supplementary Figure S5A). Figure 3B shows a non-significant decreased migration rate of BM-MNCs in all dosages, whereas some graphs shown in Figure S5A also show a non-significant increase in scratch wound coverage. However, a clear induction of endothelial cells migration after adding BM-MNC isolates cannot be observed. Moreover, in one of the six experiments, a significantly lower migration rate was observed when adding 20,000 BM-MNCs.

In this scratch wound set-up, we also studied the potential paracrine effects; conditioned medium was prepared in immune cell-suitable culture media AIMV or OptiMEM mixed 1:1 with endothelial cell culture medium EBM2 containing 0.2% serum, and added to the wounded HUVEC cultures. After 12 h, the scratch wound cultures showed no significant difference in migration rate (Figure 3C). The conditioned media in other batches of BM-MNC isolates also did not lead to any changes in the migration rate (Figure S5B).

### 2.4. No Effect of BM-MNCs on Endothelial Cell Tube Formation

**Figure 4:**
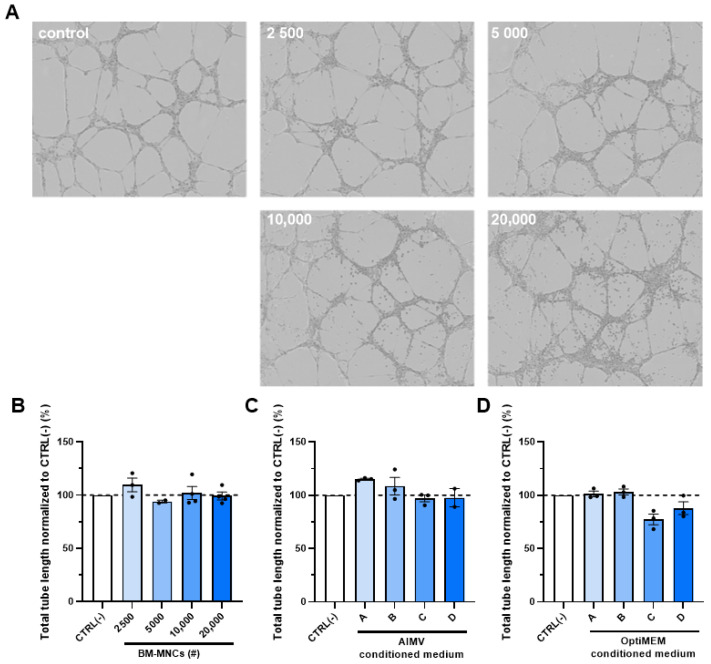
Representative microscopy photos (10×) of HUVEC tube formation with the presence of BM-MNCs (**A**), quantification of HUVEC tube formation length after treatment with (**B**) BM-MNCs (2500, 5000, 10,000, or 20,000 cells added), and after treatment with BM-MNC-conditioned medium where A, B, C, and D represent 2500, 5000, 10,000, or 20,000 BM-MNCs, respectively, in (**C**) AIMV medium or (**D**) OptiMEM medium. Data points represent three technical replicates, and are presented as mean ± SEM. Non-significant via one-way ANOVA.

The angiogenic capacity of endothelial cells in general can be studied using a Matrigel tube formation assay. Therefore, we also studied the effect of BM-MNCs on the capacity of HUVECs to form tubes in a Matrigel tube formation assay. Here, we determined the total length of the tubes formed after 12 h of incubating HUVECs with different doses of BM-MNC isolates (Figure 4 and Supplementary Figure S6). The photos clearly show the increasing BM-MNC doses that were added at t = 0, visualizable as more cells adhering to the tubular structures. Quantification of the length, however, showed no differences between the different numbers of cells added.

Quantification of the total tube length of HUVECs was performed after 8 h. The experiments were performed in triplicate with different BM-MNC isolates. The results did not show an increase in tube formation rate (Figure 4B and Supplementary Figure S6A). Figure 4B shows no differences in endothelial cell tube formation length of BM-MNCs in all dosages, which is confirmed in Figure S6A.

The indirect effects of BM-MNCs on endothelial cell tube formation length were studied by adding BM-MNC-conditioned medium to HUVECs. The conditioned medium was prepared in AIMV or OptiMEM medium, both suitable immune cell culture media to optimize the culturing conditions for the BM-MNCs. The results show no differences in the tube length (Figure 4C,D).

### 2.5. The Effect of BM-MNCs on Aortic Ring Sprouting

Aortic ring sprouting ex vivo is another very informative assay for the angiogenic potential of cells or factors. Explants of mouse aortas have the capacity to sprout and form branching microvessels ex vivo when embedded in gels of collagen. Angiogenesis in this system is driven by endogenous growth factors released by the aorta and its outgrowth in response to the injury of the dissection procedure [19]. The aortic ring assay offers many advantages over existing models of angiogenesis. Unlike isolated EC, the native endothelium of the aortic explants has not been modified by repeated passages in culture and retains its original properties. Angiogenic sprouting occurs in the presence of pericytes, macrophages, and fibroblasts, as seen during wound healing in vivo [20].

**Figure 5:**
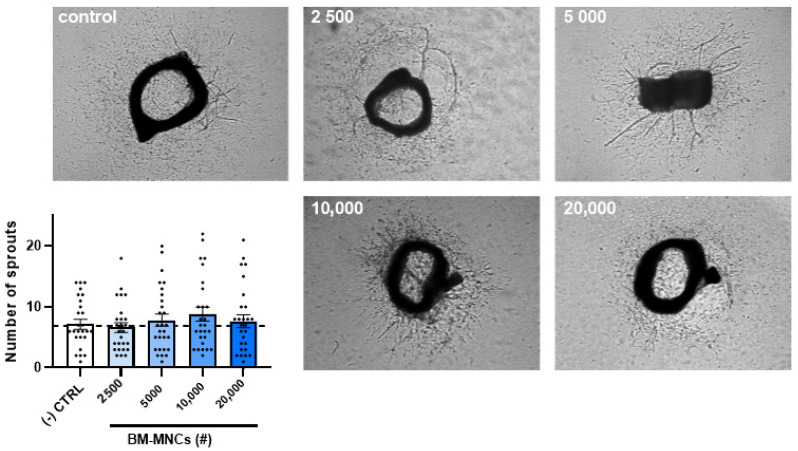
Quantification of neovessel sprouts of mice aortic rings after treatment with BM-MNCs (2500, 5000, 10,000, or 20,000 cells added). The graph is representative for 3 experiments performed with BM-MNCs isolated from 3 different bone marrow samples. Data are presented as mean ± SEM with data points in 30-fold. Non-significant via Kruskal-Wallis test.

The incubation of murine aortic rings with increasing numbers of BM-MNC isolates (2500, 5000, 10,000, or 20,000 cells added) (Figure 5) did not result in any differences in the numbers of sprouts originating from the rings. The analysis was performed after 7 days. The experiment was repeated with three different BM-MNC isolates (Figure S7).

### 2.6. BM-MNCs Release Angiogenic Cytokines

**Figure 6:**
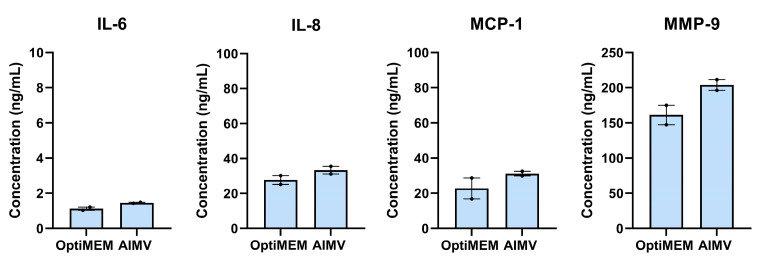
Quantification of the concentrations of IL-6, IL-8, MCP-1, and MMP-9 in OptiMEM or AIMV cell culture medium after 24 h incubation with 1.33 × 10^6^ BM-MNCs/mL. Data points represent two measurements and are presented as mean ± SEM.

Thus far, no direct and paracrine effects of BM-MNCs on endothelial cells were observed. Therefore, we were interested in determining which cytokines and factors are released by BM-MNCs. To study factors excreted by BM-MNCs, the productions of IL-6, IL-8, MCP-1, and MMP-9 were determined. In OptiMEM, BM-MNCs produced 1.1 ng/mL of IL-6, 27.7 ng/mL of IL-8, 22.8 ng/mL of MCP-1, and 161.3 ng/mL of MMP-9. In AIMV, BM-MNCs produced 1.5 ng/mL of IL-6, 33.3 ng/mL of IL-8, 31.2 ng/mL of MCP-1, and 203.9 ng/mL of MMP-9 (Figure 6).

## 3. Discussion

The current study investigated whether bone marrow cell isolates, prepared according a strict protocol defined by Rojas-Torres et al. [18], have angiogenesis-stimulating potential. The effects of these bone marrow cell isolates on endothelial cell proliferation and endothelial cell migration were subsequently analyzed. Under none of the conditions tested could any stimulatory effects be observed with various concentrations, under normoxic or hypoxic conditions, or with direct contact or paracrine effect via conditioned medium exposure. Neither effects on Matrigel tube formation nor on aortic ring sprouting could be observed after incubation with different doses of cell isolates. Due to the lack of effects observed in these models, the effects were not evaluated in other ex vivo angiogenesis models such as spheroid cultures. In an attempt to unravel the mechanism of action of these specified BM-MNC isolates that showed promising results in clinical trials, it seems that the effect is most likely not due to an induction of angiogenesis per se [10].

The effect of bone marrow-derived mononuclear cells in patients with critical limb ischemia has been studied for a couple of decades, but its effectiveness remained unclear [21,22,23]. Clinical trials showed varying results, although there are plenty of studies that showed promising effects in patients with CLTI. However, the randomized controlled trials that reported beneficial effects of BM-MNCs are of relatively low quality. Thus far, the induction of neovascularization after BM-MNC therapy has not been convincingly demonstrated. Currently, a high-quality phase III randomized controlled trial is being conducted (NCT03174522) after reporting promising phase II trial (NCT00987363) results [10]. Despite these positive clinical trial results, the supposed mechanism of action by which these injected bone marrow cells induce neovascularization remains unclear.

Interestingly, most studies using bone marrow-derived cells as a therapy did not define the composition of cell types in the product nor analyze the product for quality assessment. In this study, quality requirements were set for the BM-MNC isolates, and each cell isolate was examined to confirm an adequate product quality. Hence, the cell composition of the BM-MNCs is known, and consists of multiple mononuclear cell types in certain proportions. Setting quality requirements, and thus assessing the proportions of different cell types in the product, is an important step in understanding the mechanism of action. Furthermore, it can help to acquire knowledge about why some patients do not respond to cell therapy, which may be related to the composition of the product. It is shown that 63.63–86.87% of the BM-MNC isolates consist of CD45+ leukocytes. However, the identity of the remaining 13–36% (CD45−) of the cells is still unknown. Previous research has shown that besides CD45+ hematopoietic stem cells, bone marrow also contains a population of heterogenous CD45− nonhematopoietic tissue-committed stem cells [24]. In addition, CD45− cells in bone marrow cell fractions are of hematopoietic origin, and can be erythroid and lymphoid progenitors [25]. There is a substantial portion of CD34+ cells present in the our bone marrow cell isolates (Table 1), and CD34+ cell therapy has been shown to be one of the most promising approaches, most likely via the miR126 present in the condition medium of the CD34+ cells [26] that was reported to induce tube formation [27]. However, we were not able to demonstrate similar effects in our tube formation experiments.

Although no effects on angiogenesis were demonstrated, we showed IL-8, MCP-1, and MMP-9 to be present in the BM-MNC isolates, which are known proangiogenic factors. The role of IL-8 is widely researched in the oncologic field, where IL-8 promotes tumor angiogenesis by activating the VEGF pathway and enhancing MMP expression [13,28]. The presence of MMP-9 in the cell isolates suggests that extracellular matrix components can be degraded, which are key elements of the basement membrane surrounding blood vessels. Allowing the degradation of extracellular matrix allows endothelial cells to migrate into the surrounding tissue, starting new vessel formation [29]. MCP-1 is a chemokine that regulates the migration and infiltration of monocytes and macrophages to the site where it is released. Then, monocytes are able to differentiate into macrophages, which are important players in angiogenesis as they release factors including VEGF, MMPs, and enzymes promoting blood vessel growth by inducing endothelial cell proliferation and migration [30,31,32].

Understanding the absence of angiogenic effects of BM-MNCs on HUVECs, despite the proangiogenic chemokines that are excreted, is of great importance. A possible explanation for lacking angiogenic effects may be that the cell products were produced from bone marrow obtained from healthy donors. Although the BM-MNC isolates manufactured in this study fulfilled all quality criteria, BM-MNC isolates manufactured from CLTI patients (REX-001) suffering from type 2 diabetes mellitus may have a different composition and characteristics. Furthermore, the in vitro set-up only involved physiological HUVECs, while in the pathophysiologic situation of patients with CLTI dysfunctional endothelial cells are involved many more cells, chemokines, and inflammatory markers [33].

In this study, we studied the effects on HUVECs in normoxic and hypoxic environments, because endothelial cells in patients with CLTI suffer from hypoxia-induced endothelial cell dysfunction [34]. In addition, multiple culture media were used in the experiments to optimize the culturing conditions for both the BM-MNCs and the HUVECs together. Despite our efforts to unravel their effects on endothelial cells, one should bear in mind that other cell types, including smooth muscle cells, monocytes, and pericytes, are involved in angiogenesis and arteriogenesis. These cell types were not involved in our experiments, which is a limitation in our approach. However, REX-001 was studied in a murine model with the presence of all of the cell types involved, and improvement in revascularization and ischemic reperfusion was concluded [18]. Future fundamental biological studies should focus on identifying effects on these cell types. Moreover, there is a need for strategies to identify and augment the homing, survival, and effectiveness of the injected cells.

We believe that future clinical studies directed at cell therapeutic approaches to relieve CLI in patients should be based on a clear mechanism of action to avoid more disappointing clinical trial results.

## 4. Materials and Methods

### 4.1. BM-MNC Isolates Manufacturing

BM-MNCs were isolated from the heparinized bone marrow of healthy human donors (Hemacare, Charles-River) using a scaled-down density gradient. In short, the bone marrow was filtered using a 180 µm filter, and a sample of the filtered bone marrow was used for hematology analysis. The filtered bone marrow was then separated using Ficoll-Paque 1.077, and the upper layer, including the plasma and low-density cells, was isolated. These cells were washed twice with isotonic saline solution containing 2.5% human serum albumin. Finally, the BM-MNCs were resuspended in Ringer’s lactate solution containing 2.5% *w*/*v* glucose and 1% *w*/*v* HSA.

The filtered bone marrow and the final product were both measured in a Sysmex XP-300 hematology analyzer. The obtained amounts of leukocytes, erythrocytes, and thrombocytes were used to calculate the leukocyte recovery percentage, and the percentages of erythrocyte and thrombocyte depletion.

### 4.2. Flow Cytometric Analysis

Flow cytometry was performed on the isolated BM-MNC batches. The possible remaining erythrocytes were lysed in ACK lysis buffer (A1049201, Thermo Fisher, Waltham, MA, USA) and washed twice with PBS supplemented with 0.1% heat-inactivated fetal bovine serum. The conjugated antibodies to human CD45 (HI30, 1/200, 50 µg/mL, BioLegend, San Diego, CA, USA), CD3 (OKT3, 1/125, 30 µg/mL, BioLegend), CD4 (OKT4, 1/400, 150 µg/mL, BioLegend), CD8a (HIT8a, 1/300, 50 µg/mL, BioLegend), CD19 (HIB19, 1/200, 50 µg/mL, BioLegend), CD14 (M5E2, 1/400, 400 µg/mL, BioLegend), CD16 (3G8, 1/800, 0.5 mg/mL, BioLegend), CD56 (IgG κ, 1/200, 200 µg/mL, BioLegend), and CD34 (581, 1/100, 50 µg/mL, BioLegend) were incubated on ice for 30 min. 7-AAD viability staining solution (1/800, 50 µg/mL, BioLegend) was used as a viability marker. The flow cytometric acquisition was performed on an Aurora 3 Laser (Cytek, Fremont, CA, USA). The flow cytometry data were analyzed using FlowJo V10.1 software (BD).

### 4.3. Cell Culture of Human Umbilical Vein Endothelial Cells (HUVECs)

Human umbilical vein endothelial cells (C2519AS, Lonza, Basel, Switzerland) were cultured in plates coated with 0.1% gelatin in PBS in EBM-2 culture medium (CC-3156, Lonza, Basel, Switzerland) supplemented with EGM-2 SingleQuots (CC-4176, Lonza, Basel, Switzerland), and were between passages 3 and 4. The cells were incubated at 37 °C in a humidified 5% CO_2_ environment.

### 4.4. BM-MNC Conditioned Medium

To prepare the conditioned media, 1.33 × 10^6^ BM-MNCs/mL were incubated for 24 h in EBM-2, OptiMEM (Gibco, Billings, MT, USA), and AIMV (Gibco) culture media. The conditioned media were stored at −80 °C, and were thawed and diluted for use in the experiments.

### 4.5. MTT Assay

The cell proliferation (*n* = 4 experimental replicates) of the HUVECs was determined using MTT assays. A volume of 100 µL of HUVECs (4000 cells/well) were plated in 96-well plates and cultured until approximately 80% confluency was reached in complete endothelial cell culture medium. The medium was then replaced by endothelial cell low-serum medium containing 0.2% FBS for 24 h. Subsequently, the medium was replaced by BM-MNC treatments consisting of low-serum media containing 2500, 5000, 10,000, or 20,000 BM-MNCs. After 24 h of incubation, 10 µL of MTT (Thiazolyl, blue tetrazolium bromide, Sigma M5655) was added per well. The cells were incubated for 4 h, after which 75 µL of each well was discarded and replaced by 75 µL of isopropanol/0.1 M hydrogen chloride. The plates were incubated at room temperature on a platform shaker until dissolution of the formazan crystals was observed. Thereafter, the absorbance was read at 570 nm on a Cytation5 spectrophotometer (BioTek, Winooski, VT, USA), and the data were obtained using BioTek Gen5 software. The obtained mitochondrial metabolic activity data were quantified as a representative measure of cell proliferation.

### 4.6. Scratch Wound Healing Assay

For the scratch wound healing assays (*n* = 6 experimental replicates), HUVECs were plated on IncuCyte Imagelock 96-well plates (BA-04856, Sartorius AG, Goettingen, Germany) and cultured until approximately 90% confluence was reached in complete culture medium, as previously mentioned. The medium was then replaced by EBM-2 Basal medium supplemented with 2% FBS (SingleQuotsTM Supplements, CC-4176, Lonza) and 1% GA-1000 (SingleQuotsTM Supplements, CC-4176, Lonza). After 24 h, a scratch wound was introduced using the Incucyte Woundmaker Tool (4563, Sartorius AG, Goettingen, Germany), and different amounts of BM-MNCs were added in EBM-2 Basal medium supplemented with 2% FBS and 1% GA-1000. The plates were incubated in the IncuCyte S3, and pictures were taken after 12 h. The percentage of scratch wound closure after 12 h was calculated by measuring the difference in the wound surface at baseline and the wound surface after 12 h using the Wound Healing Tool of ImageJ.

### 4.7. Tube Formation Assay

HUVECs were seeded in a 6-well plate in EBM-2 culture medium supplemented with SingleQuots until they became confluent. The medium was replaced with low-serum medium for 24 h. Then, a 96-well plate was coated with 45 µL/well of Geltrex basement membrane matrix (A1413202, ThermoFisher, Waltham, MA, USA). Suspensions of HUVECs at a concentration of 250,000 cells/mL and different concentrations of BM-MNCs or BM-MNC-conditioned medium were prepared and seeded in the coated 96-well plate. The plate was incubated in IncuCyte S3, and pictures were taken every 2 h for 24 h. The analysis was performed using ImageJ at t = 8 h.

### 4.8. Aortic Ring Assay

The 8-week-old mice were sacrificed, the aortas were resected, and the surrounding fat and branching vessels were removed. The aortas were cut in <1 mm rings, and were overnight incubated at 37 °C in a humidified 5% CO_2_ environment in OptiMEM supplemented with 1% penicillin/streptomycin. A 96-well plate was coated with 75 µL of collagen matrix (Collagen (Type 1, Merck Sigma-Aldrich, Millipore, Burlington, MA, USA) in DMEM (ThermoFisher, Waltham, MA, USA), pH adjusted with 5N NaOH), and then one aortic ring was added per well. After one hour, the collagen was solid and 150 µL of OptiMEM supplemented with 2.5% FBS, 1% penicillin–streptomycin solution (Cytiva, HyClone Laboratories, North Logan, UT, USA), 10 ng/mL of mouse VEGF (BioLegend, San Diego, CA, USA), and different amounts of BM-MNCs were added to each ring, with 20 or 30 rings per condition. After a total of 7 days of incubation at 37 °C in a humidified 5% CO_2_ environment, with a medium replacement after 3 days, pictures of each aortic ring were taken using live phase-contrast microscopy (Axiovert 40C, Carl Zeiss, Oberkochen, Germany). The number of sprouts were counted manually.

### 4.9. ELISA

The bone marrow-derived mononuclear cells were plated at 1.33 × 10^6^ cells/mL for 24 h to prepare the conditioned medium. After 24 h, the supernatant was stored at −20 °C. The IL-6, IL-8, MCP-1, and MMP-9 concentrations were determined via ELISA, according to the protocol (BD Biosciences, San Jose, CA, USA) in the supernatant of the BM-MNCs.

### 4.10. Statistical Analysis

Differences in the continuous variables between groups were statistically assessed using one-way ANOVA or Kruskal–Wallis tests in Graph Pad Prism 8 software. The data are represented as means ± SEM. The significance was set at *p* < 0.05.

## 5. Conclusions

In this study, no effect from human bone marrow cell isolates on the angiogenic behavior of experimental human endothelial cells (HUVEC) could be demonstrated. Our research holds significant relevance, as it addresses the shortage of supporting evidence regarding the effects of BM-MNCs on cultured endothelial cells.

## Figures and Tables

**Table 1:** The ranges of process performance indicators and cell populations from six times manufacturing the BM-MNC isolate.

Process Performance Indicator	BM-MNCs Relative to Bone Marrow (%)	Acceptance Criteria (%)
Leukocyte recovery	13.05 *–22.28	>15%
Erythrocyte depletion	99.91–100	>96%
Thrombocyte depletion	95.79–100	>60%
**Cell population**	**BM-MNCs (%)**	
Viability	99.50–99.90	>80%
Leukocytes (CD45+) ^1^	63.63–86.87	-
B lymphocytes (CD19+)	1.08–4.63	-
T lymphocytes (CD3+)	4.84–9.74	-
CD4+ T lymphocytes	2.52–4.68	-
CD8+ T lymphocytes	1.36–3.42	-
Granulocytes (CD16+, CD14−)	68.23–84.40	>30%
Monocytes (CD14+)	2.76–10.50	-
CD34+ leukocytes	1.34–3.59	>0.1%

* This cell isolate was not used in experiments. ^1^ of single viable cells.

## Data Availability

Data can be made available upon request.

## Supplementary Materials

The following supporting information can be downloaded at: https://www.mdpi.com/article/10.3390/ijms241813822/s1.
