# Supplementary material for: Exploring the Effects of Human Bone Marrow-Derived Mononuclear Cells on Angiogenesis In Vitro
Source: Int J Mol Sci. 2023 Sep 7;24(18):13822. doi: 10.3390/ijms241813822 (PMC10531254; doi:10.3390/ijms241813822)
Supplement: Supplementary file 1 [file ijms-24-13822-s001.zip › ijms-2576685-supplementary.pdf]

International Journal of *Molecular Sciences*

Appendix with Supplemental Figures

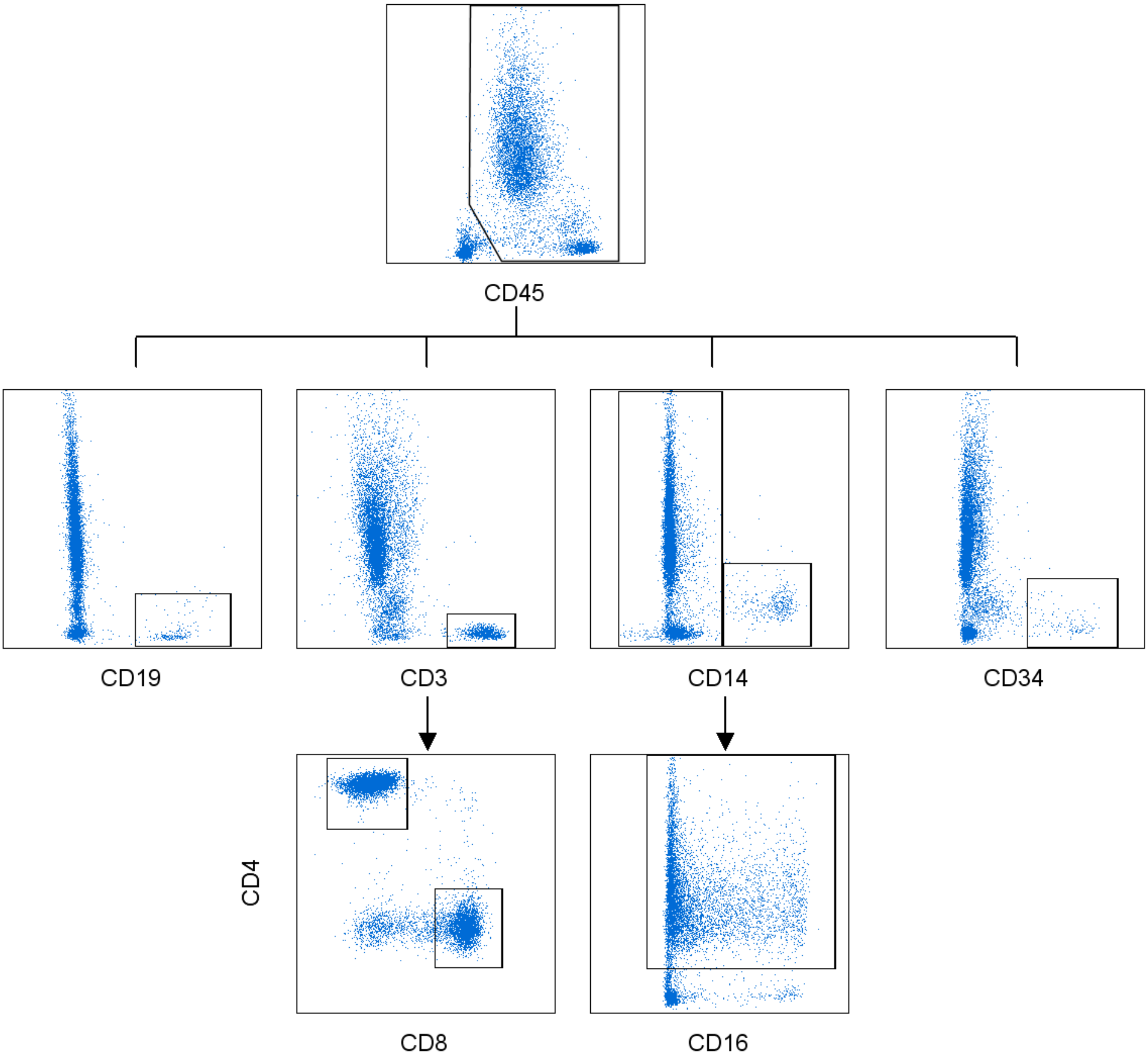

Supplemental Figure S1. Gating strategy for REX-001. Cells were selected from side scatter-area vs FSC-H, then single cells were selected from FSC-H vs FSC-A and subsequently the viable cells were selected using 7AAD and FSC-H. The CD45+ cells were selected from the viable cells and subsequently CD 19+, CD3+, CD14+/- and CD34+ cells were selected from the CD45+ cells. The CD3+ cells were further specified in CD4+ and CD8+ cells. The CD14- selection was further specified for CD16+ cells.

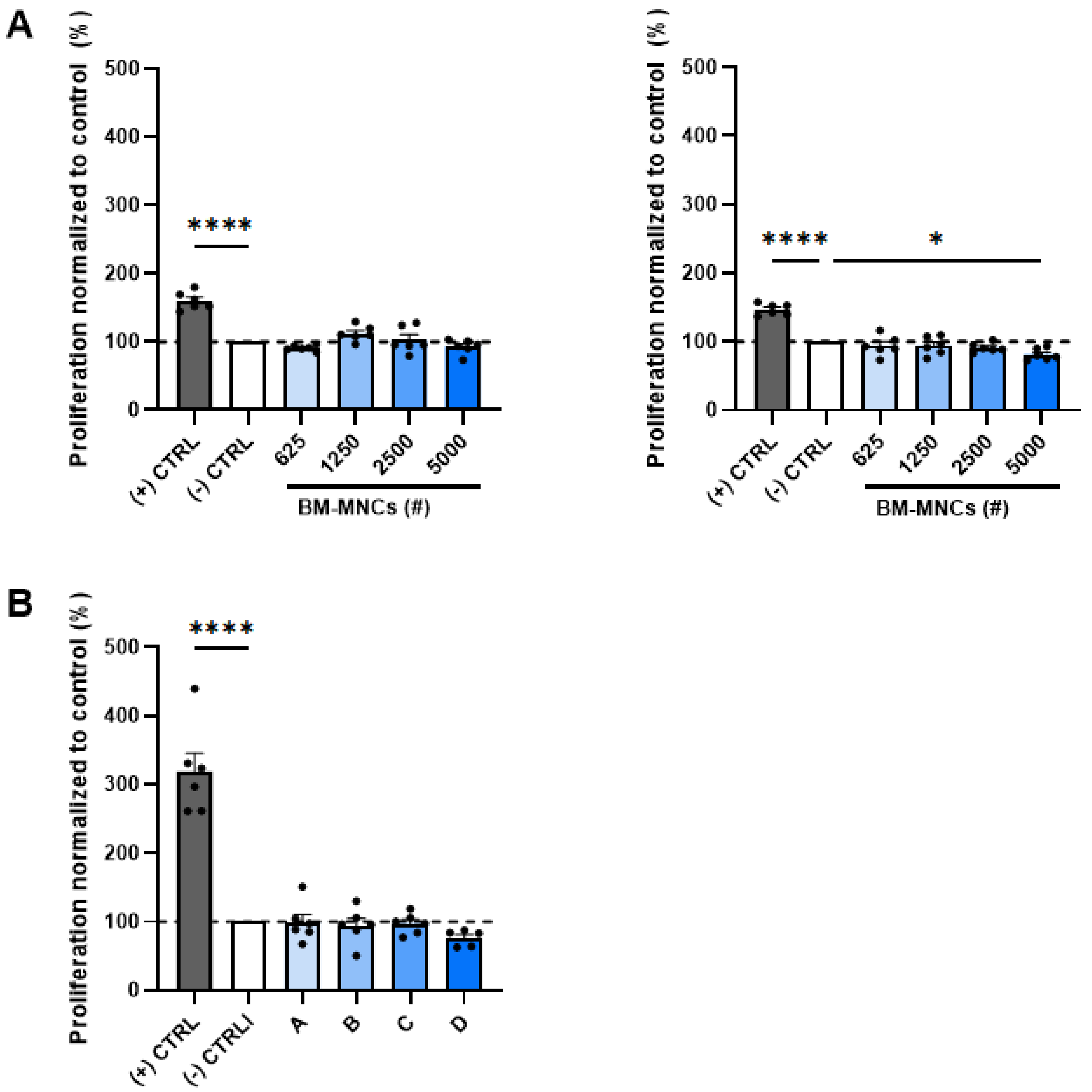

Supplemental Figure S2. Quantification of HUVEC proliferation after treatment with either **(A)** BM-MNCs (625, 1250, 2500 or 5000 cells added) or with **(B)** BM-MNC conditioned medium A, B, C and D respectively representative for 2500, 5000, 10.000 or 20.000 BM-MNCs. Graph S2A shows 2 experiments performed with BM-MNC isolates manufactured from 2 different bone marrow samples. Graph S2B is an experiment performed with BM-MNC conditioned medium. Data are presented as mean ± SEM with datapoints in sextuplicate. *$p < 0.05$, ***$p < 0.001$, ****$p \leq 0.0001$ by One-Way ANOVA.

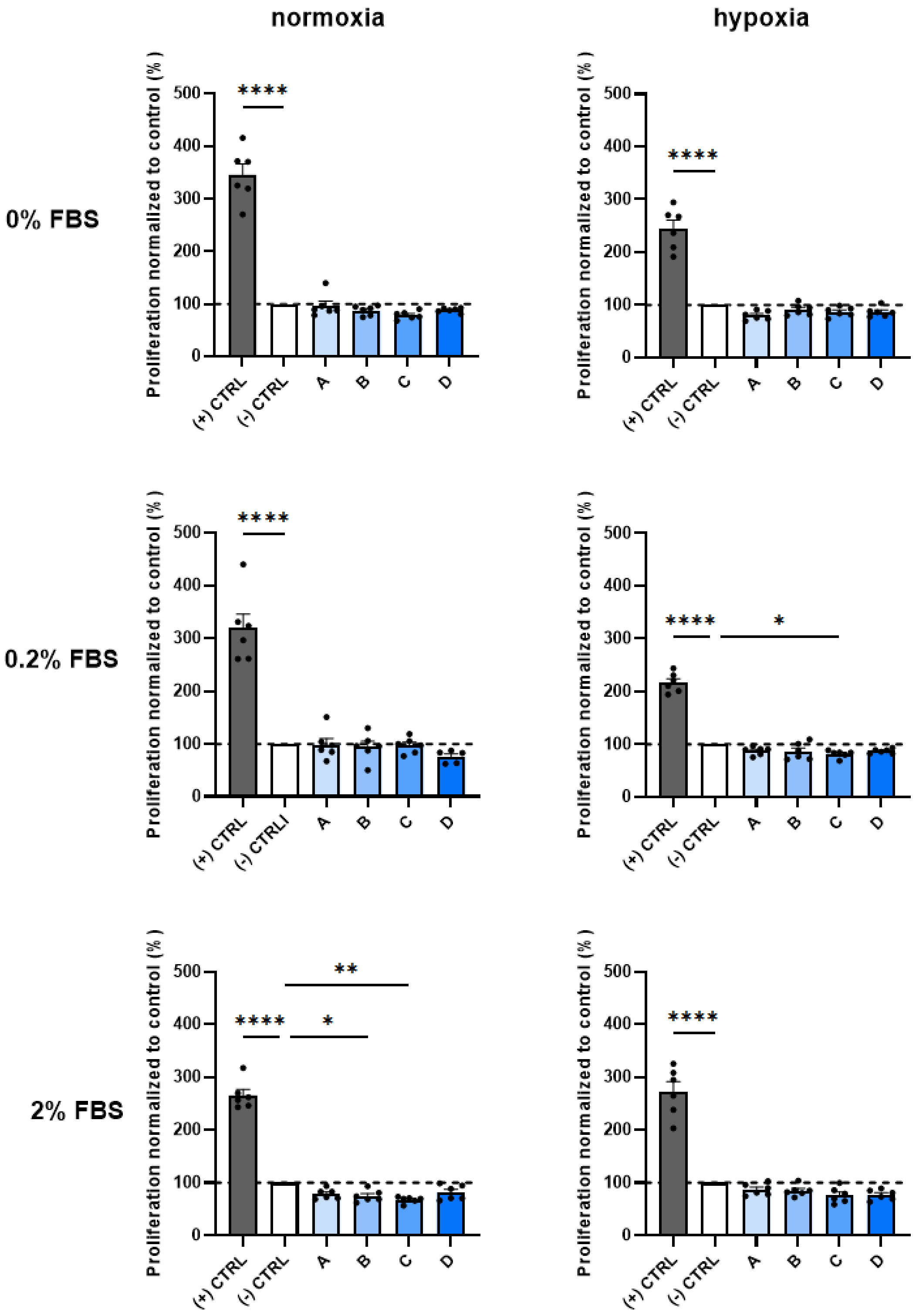

Supplemental Figure S3. Quantification of HUVEC proliferation after treatment with BM-MNC conditioned medium in EBM-2 medium without FBS, with 0.2% or 2% PBS in normoxic or hypoxic conditions. A, B, C and D represent conditioned medium with respectively 2500, 5000, 10.000 or 20.000 BM-MNCs. The graphs shown are a representative of 2 experiments. Data are presented as mean ± SEM with datapoints in sextuplicate. **p* < 0.05, ***p* < 0.01, ****p* < 0.001, *****p* ≤ 0.0001 by One-Way ANOVA.

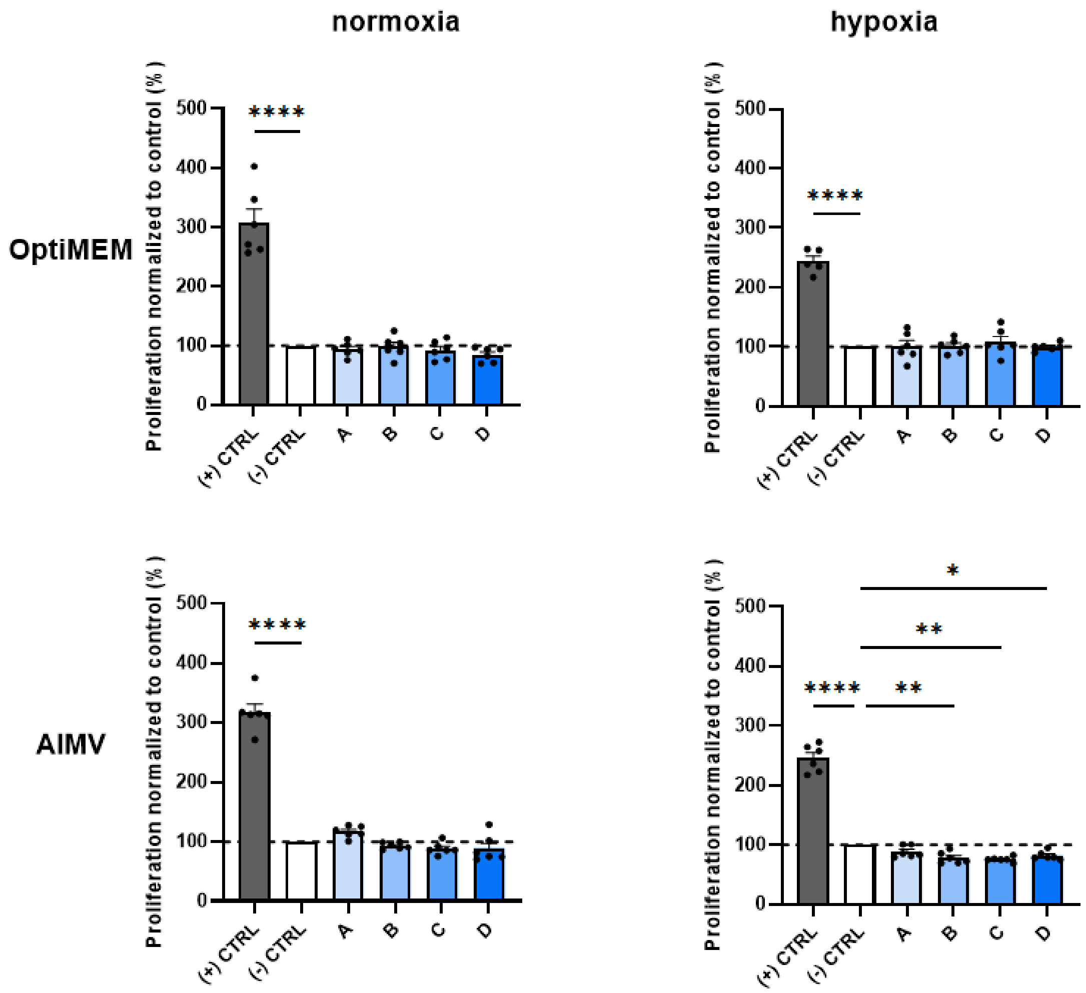

Supplemental Figure S4. Quantification of HUVEC proliferation after treatment with BM-MNC conditioned medium in OptiMEM or AIMV culture medium in normoxic or hypoxic conditions. A, B, C and D represent conditioned medium with respectively 2500, 5000, 10.000 or 20.000 BM-MNCs. The graphs shown are a representative of 2 experiments. Data are presented as mean ± SEM with datapoints in sextuplicate. *$p < 0.05$, **$p < 0.01$, ****$p \leq 0.0001$ by One-Way ANOVA.

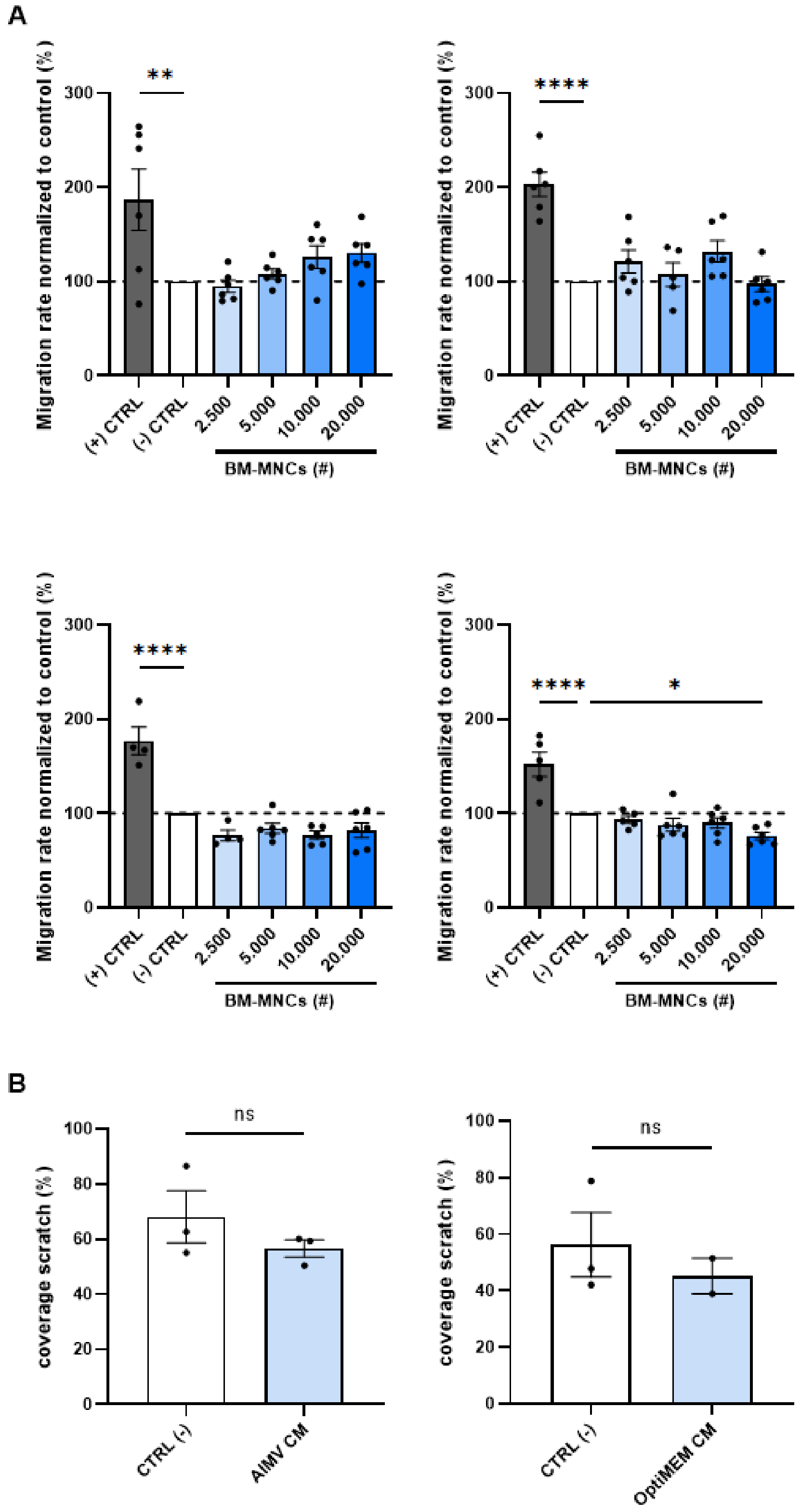

Supplemental Figure S5. Quantification of HUVEC scratch-wound healing after treatment with BM-MNCs (2.500, 5.000, 10.000 or 20.000 cells added) **(A)** or after treatment with BM-MNC conditioned medium (CM) of AIMV or OptiMEM **(B)**. These graphs show the additional 5 experiments performed with BM-MNC isolates manufactured from 5 different bone marrow samples and conditioned medium manufactured from 1 bone marrow sample, respectively. Data are presented as mean ± SEM with datapoints in sextuplicate **(A)** or triplicate **(B)**. *ns* non-significant, *$p \leq 0.05$, **$p \leq 0.01$, ***$p \leq 0.001$, ****$p \leq 0.0001$ by One-Way ANOVA.

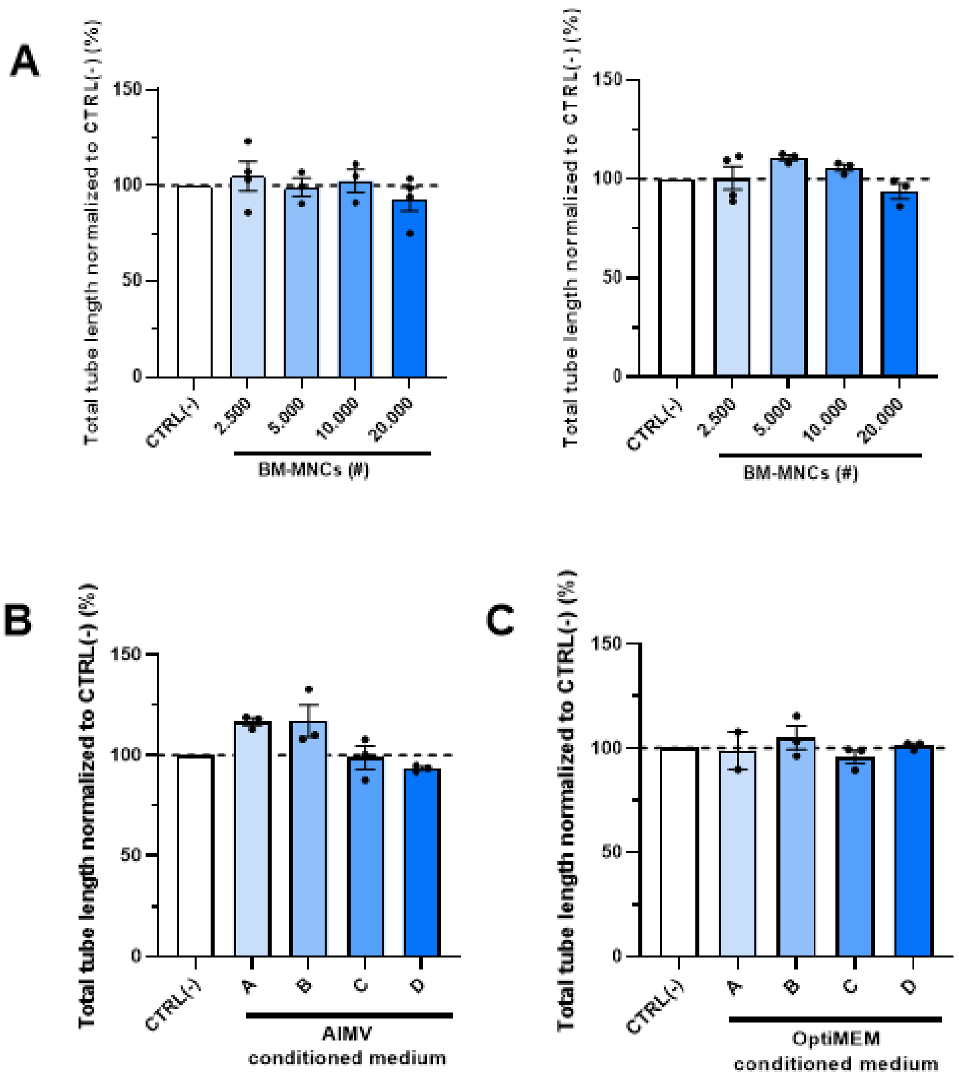

Supplemental Figure S6. Quantification of HUVEC tube formation length with the presence of BM-MNC isolates of two different donor batches **(A)**, after treatment with **(B)** BM-MNC conditioned AIMV medium or **(C)** BM-MNC conditioned OptiMEM medium. In conditioned medium A, B, C and D respectively represent 2500, 5000, 10.000 or 20.000 BM-MNCs, the amount of cells that were used to produce the conditioned medium. Datapoints represent four(A) or three(B, C) technical replicates, and are presented as mean ± SEM. Non-significant, by One-Way ANOVA.

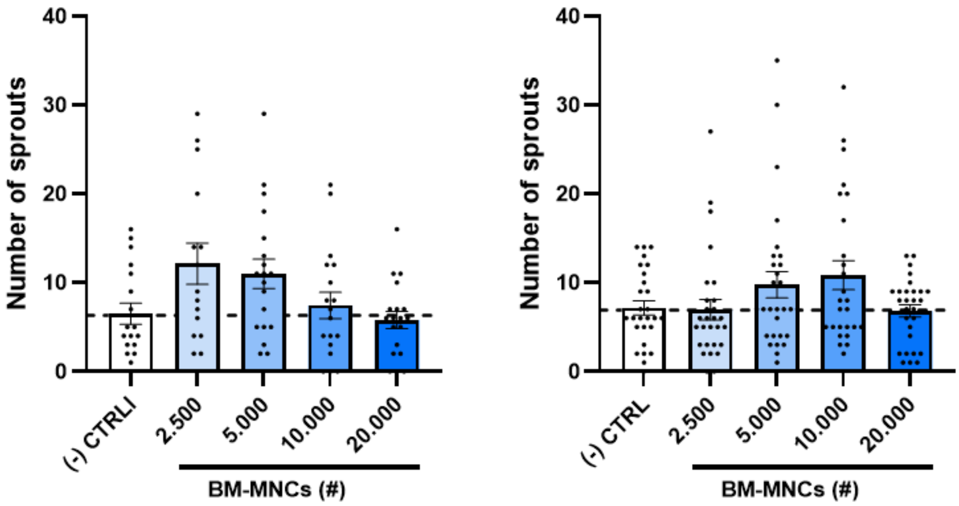

Supplemental Figure S7. Quantification of neovessel sprouts of mice aortic rings after treatment with BM-MNCs (2.500, 5.000, 10.000 or 20.000 cells added). Data are presented as mean ± SEM with datapoints in 30-fold. Non-significant by Kruskal-Wallis test.
